# From Marshes to Mines: Germination and Establishment of *Crinum bulbispermum* on Gold Mine Tailings

**DOI:** 10.3390/plants14152443

**Published:** 2025-08-07

**Authors:** Vincent C. Clarke, Sarina Claassens, Dirk P. Cilliers, Stefan J. Siebert

**Affiliations:** 1 Unit for Environmental Sciences and Management, North-West University, Potchefstroom 2520, South Africastefan.siebert@nwu.ac.za (S.J.S.); 2 School of Molecular and Life Sciences, Curtin University, Bentley, WA 6102, Australia

**Keywords:** grassland, geophyte, restoration, mine tailings

## Abstract

The growth potential of *Crinum bulbispermum* was evaluated on gold mine tailings. The primary objectives were to model the species’ climatic niche in relation to gold mining regions, assess its germination success on tailings, and compare seedling survival and growth on tailings versus other soil types. Species distribution modelling identified the South African Grassland Biome on the Highveld (1000+ m above sea level), where the majority of gold mines are located, as highly suitable for the species. Pot trials demonstrated above 85% germination success across all soil treatments, including gold mine tailings, indicating its potential for restoration through direct seeding. An initial seedling establishment rate of 100% further demonstrated the species’ resilience to mine tailings, which are often seasonally dry, nutrient-poor, and may contain potentially toxic metals. However, while *C. bulbispermum* was able to germinate and establish in mine tailings, long-term growth potential (over 12 months) was constrained by low organic carbon content (0.11%) and high salinity (194.50 mS/m). These findings underscore the critical role of soil chemistry and organic matter in supporting long-term plant establishment and growth on gold tailings. Building on previous research, this study confirms the ability of this thick-rooted geophyte to tolerate chemically extreme soil conditions. *Crinum bulbispermum* shows promise for phytostabilization and as a potential medicinal plant crop on tailings. However, future research on microbial community interactions and soil amendment strategies is essential to ensure its long-term sustainability.

## 1. Introduction

Ancient old-growth grassland is the second largest biome in South Africa and provides essential ecosystem services such as medicinal and food plants, thatch grass, and carbon sequestration [1]. Despite its ecological and economic importance, South African grasslands remain poorly conserved, facing significant threats from mining, agriculture, overgrazing, and overharvesting, which have resulted in a 35% transformation [2]. Mining and agricultural activities in the Grassland Biome often result in soil degradation, characterized by acidic pH, elevated concentrations of potentially toxic metals and metalloids, and low organic carbon content. These conditions impede plant establishment and delay ecosystem recovery [3]. South Africa’s economy is heavily reliant on mining, with over 500 active mines, the majority of which are gold mines. Currently, 89 gold mines are located primarily in Gauteng, with additional operations in the North West and Free State Provinces (Minerals Counsil SA) [4].

Gold mining generates tailings, a type of fine-grained waste material with a high water content, facilitating transport to slimes dams [5,6]. Tailings are distinct from other mining waste such as waste rock, slag, and leaching pads. They are composed of milled rock, sand, clay, chemical residues from metallurgical processes, and trace elements including arsenic (As), cadmium (Cd), chromium (Cr), lead (Pb), and mercury (Hg), all which can pose significant environmental and health risks [7]. These tailings may be mixed with process water left over from the extraction and treatment of ores or concentrates [6]. Arsenic, commonly used in the processing of gold and other metals, is a notable contaminant in mine tailings [8]. The environmental consequences of gold tailings include the accumulation of heavy metals, acid rock drainage, dust emission, and surface runoff, with substantial impacts on agricultural land, human well-being, and ecosystem functioning in South Africa [9].

The restoration of grasslands plays a pivotal role in addressing global environmental challenges by conserving biodiversity, mitigating and adapting to climate change, and supporting human livelihoods [10]. Despite being frequently mischaracterized as degraded remnants of agriculture or deforestation with limited conservation value, natural and semi-natural grasslands are ecologically significant and widespread [11]. At finer scales, grasslands are among the most species-diverse terrestrial ecosystems, particularly in temperate regions. Globally, grasslands cover approximately 30% of terrestrial surfaces and are estimated to sequester around 12% of terrestrial carbon, primarily stored below ground, where it persists longer than in many forests [12]. In global climate mitigation strategies, grasslands serve as vital complements to forests and other high-carbon ecosystems and should be prioritized on the ecological restoration agenda [13,14].

Geophytes are integral components of grassland ecosystems but are often overlooked in management and restoration efforts. Although abundant and providing various ecosystem services, little information exists regarding the potential use of geophytes in rehabilitation [15], despite their widespread distribution and ease of propagation. The genus *Crinum* [16], which exhibits a pantropical distribution centered in Africa, includes species common in grassland habitat such as the South Africa Highveld, where they inhabit marshy black clay or sandy soils [17]. These geophytes survive adverse environmental conditions through dormancy, supported by large underground storage organs. Their resilience to fire, frost, and grazing makes them promising candidates for grassland restoration efforts [17]. As a keystone species, they may help to re-establish lost ecological processes and restore vital ecosystem services [18].

The genus *Crinum* (*Amaryllidaceae*) comprises approximately 130 species [19], with its center of diversity in sub-Saharan Africa [20]. Many species have a rich complement of medicinal and cultural uses and have yielded over 170 pharmacologically active compounds, primarily alkaloids [21,22]. As a result, *Crinum* species are increasingly threatened by overharvesting for the medicinal plant trade. Species commonly found at ‘muthi’ markets (places selling traditional medicine) include *Crinum bulbispermum*, *C. macowanii*, *C. moorei*, and *C. stuhlmannii* [17].

**Figure 1:**
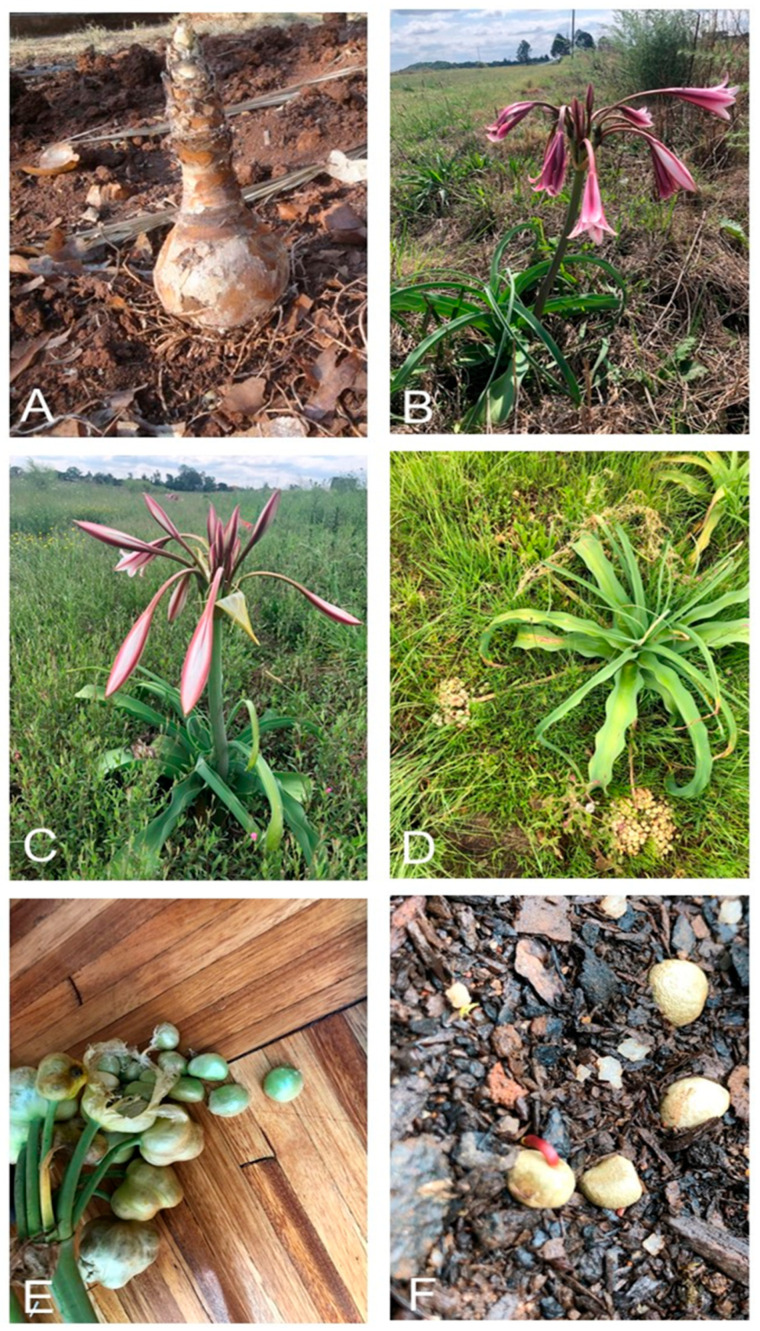
Focal species of this study, *Crinum bulbispermum*: bulb (**A**), flowers (**B**), buds (**C**), seed dispersal (**D**), seeds (**E**), and germination showing red radicle (**F**). Photos: Charl Clarke.

*Crinum bulbispermum* (Figure 1) was selected as our focal species for this study due to its declining populations resulting from overharvesting and habitat loss due to ploughing, as well as its known tolerance to potentially toxic metals [23]. Its medicinal value offers additional economic potential, and its large underground storage organ (up to 15 kg) may enhance ecosystem functionality through carbon sequestration. To address ongoing grassland degradation amidst numerous overarching threats to biodiversity, the identification and distribution modelling of ecologically beneficial keystone species such as *C. bulbispermum* is increasingly critical for informing restoration strategies [24].

When assessing a species for restoration purposes, it is important to consider the habitat it occupies and the climatic range of its distribution. A core concept in ecology is knowing the distribution of species [25] and the relationship between a species and its environment [26]. To support this, numerous species distribution models (SDMs) or ecological niche models (ENMs) have been developed since the 1980s [25]. Both model types estimate the habitat suitability for specific species, define environmental tolerances of the species, and predict species responses of the species to climate change or other disturbances [27,28]. Currently, no spatial distribution models exist that correlate the distribution of *C. bulbispermum* with environmental factors. This represents a significant gap in our understanding of the species’ environmental preferences and its potential for restoration applications.

The aim of this study was to evaluate the potential of *C. bulbispermum* for use in the restoration of gold mine tailings on the Highveld of South Africa. The specific objectives were as follows: (1) to identify the preferred environmental conditions of *C. bulbispermum* by mapping its natural distribution and modelling its climatic niche in regions where gold mine tailings occur; (2) to assess the germination success of the species in gold mine tailings; and (3) to compare seedling survival and growth in pot trials across gold tailings and other soil types, using plant productivity as an indicator of restoration potential. The outcome of this study was to generate a restoration profile for *C. bulbispermum*, detailing its environmental preferences and growth potential on tailings with marshy conditions [29]. Such a profile would support its inclusion in tailored seed mixes for restoration initiatives and enhance the establishment of native geophytic forbs—often overlooked in traditional grass-dominated restoration strategies [30].

## 2. Results

### 2.1. Spatial Distribution

**Figure 2:**
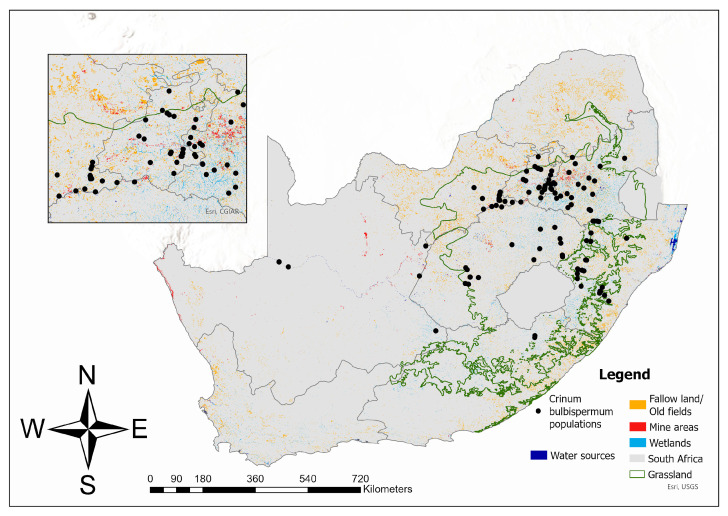
Distribution of *Crinum bulbispermum* populations in relation to mines and fallow fields inside and outside the Grassland Biome in South Africa. The inset indicates the region in and around Gauteng, where the majority of populations and gold mines are concentrated.

A presence-based distribution map of *C. bulbispermum* depicts the species’ geographical range and population densities, revealing several key patterns. More than 84% of the known locations—153 out of the 182 currently recorded—are located within the Grassland Biome. Populations are particularly concentrated in the eastern regions of South Africa, especially in the provinces of Gauteng and Mpumalanga, as well as along the Vaal River (Figure 2). In contrast, the western regions exhibit few to no populations, although there is a notable association with the Orange River in the Northern Cape. This distribution pattern suggests that the species prefers the wetter, eastern Highveld region or moist floodplains of large perennial rivers in otherwise semi-arid regions.

*Crinum bulbispermum* populations occurring along the Vaal River are frequently found in close proximity to agricultural fields and mining areas (Figure 2). This suggests that the natural distribution of *C. bulbispermum* often overlaps with heavily disturbed landscapes, indicating a degree of ecological adaptation to both local climatic conditions and anthropogenically altered soils.

### 2.2. Species Distribution Modelling

**Figure 3:**
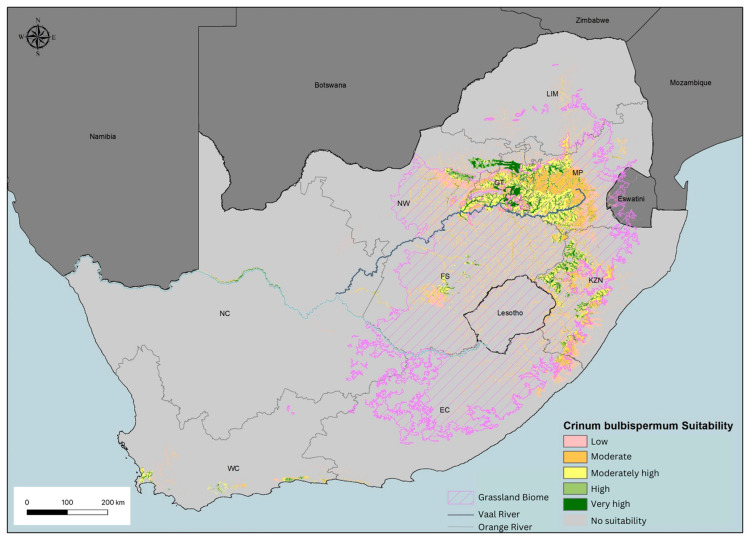
The species distribution model for *Crinum bulbispermum* predicting the majority of suitable habitat for the species in the Grassland Biome of South Africa.

Building on the current distribution of *C. bulbispermum* (Figure 2), a species distribution model (SDM) was developed to predict potential areas where the species could thrive under preferred environmental conditions (Figure 3). This model integrates observed population data with key environmental variables (Table 1), enabling the identification of regions that are ecologically suitable for the species but may currently lack established populations.

The SDM identified several areas with high to very high habitat suitability beyond the species’ known range (Figure 3), particularly along major rivers in the Northern Cape and widely within the moist grasslands of the eastern provinces. These predictions suggest that *C. bulbispermum* populations could be sustained in these areas, provided that pollination and dispersal requirements are met (factors not considered further in this study).

Vegetation type (Veg), annual precipitation (bio 12), and proximity to rivers (Distall) emerged as the most influential variables determining the distribution of *C. bulbispermum* compared to other environmental factors (Table 1). These findings support the previously described habitat preferences of the species, which is moist grassland in regions with high rainfall or floodplains adjacent to large perennial rivers (11).

The distribution of *Crinum bulbispermum* was closely associated (Figure 3) with the Vaal River system, as evidenced by the snake-like pattern of occurrence along this prominent geographical feature within the Grassland Biome and extending westward. *Crinum bulbispermum* is commonly known as the Vaal River lily, underscoring the species’ strong ecological affinity for riparian environments. A comparison of Figure 2 and Figure 3 reveals an extensive network of mines within the Vaal River basin. This spatial overlap not only reinforces the species’ alignment with the Grassland Biome but also highlights the ecological importance of riparian habitats in supporting its distribution within mining areas.

### 2.3. Standard Germination Tests

**Figure 4:**
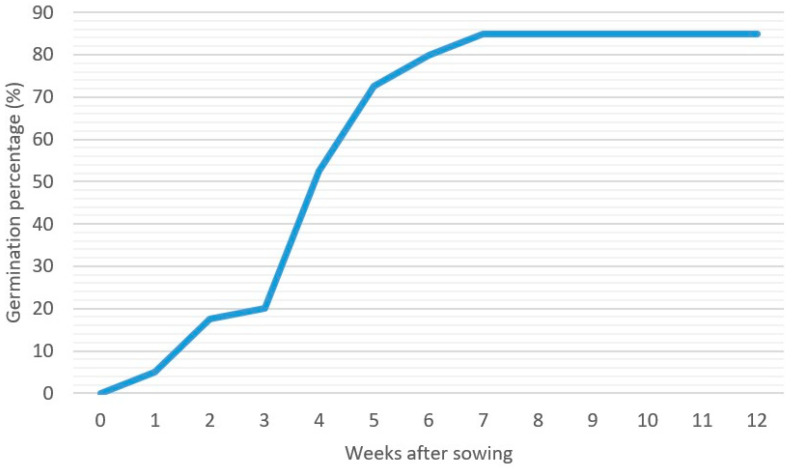
Germination percentage (%) per week after sowing. Derived from mean germination percentage of ten pots (10 seeds per pot). At daily photosynthetically active radiation between 600 and 800 µmol m^−2^ s^−1^.

A standard germination test (SGT) of seed germinability was conducted to determine the proportion of seeds capable of producing normal seedlings under optimal conditions. Germination percentages were recorded weekly after sowing (Figure 4). Germination started in week 1, with the maximum germination percentage reached at week 7; no additional germination was observed thereafter. The average germination rate was 85.5%, indicating that the seeds were non-dormant and exhibited high viability.

**Figure 5:**
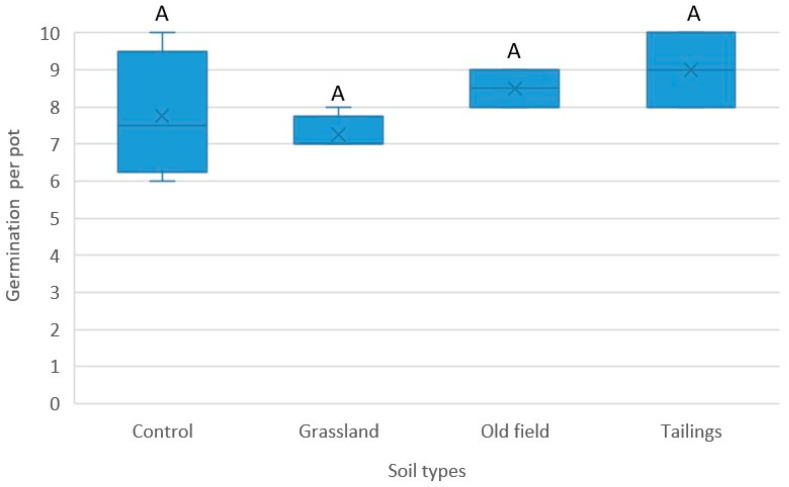
Mean germination per pot of *Crinum bulbispermum* in different soil treatments. Ten seeds per pot (n = 4). Capital letter A indicates there was no significant difference (*p* > 0.05) between the soil treatments; whiskers extending from the boxes indicate variability outside the upper and lower quartiles.

Germination rates were compared between soil treatments (Figure 5). One-way ANOVA detected no significant differences in germination percentage among soil types (*F*(3, 12) = 2.39, *p* = 0.12). This indicated that soil characteristics had no significant effect on the germination of seeds on top of the soil.

### 2.4. Establishment

Establishment success was assessed based on seedling survival and leaf length across soil treatments. Soil type had no effect on seedling survival after 12 months, with 100% survival observed across all treatments, including mine tailings.

**Figure 6:**
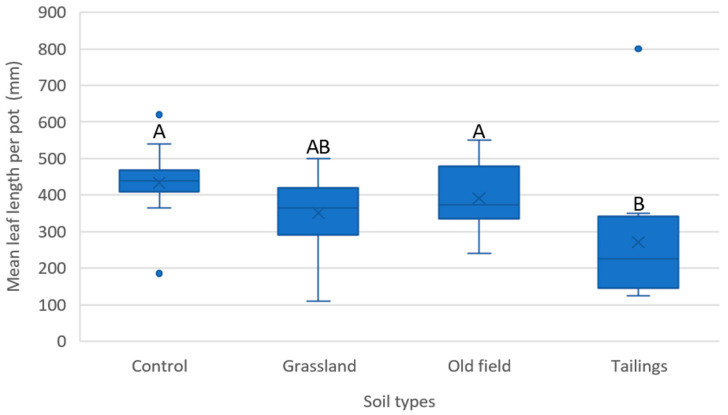
Mean leaf length for each soil type after 12 months. Ten plants per pot and four pots per treatment (n = 40). Different capital letters indicate significant statistical differences (*p* < 0.05); whiskers extending from the boxes indicate variability outside the upper and lower quartiles; dots represent outliers.

Leaf length was measured monthly for seedlings in each soil treatment. After 12 months, a statistically significant difference in leaf length was observed among soil types (*F*(3, 44) = 3.43, *p* = 0.025). A Bonferroni post hoc test revealed that only the mean leaf length of seedlings grown in mine tailings differed significantly (*p* < 0.05) from those in the control and old field soil. Leaf growth, used here as a proxy for productivity, was more robust in the control and disturbed old field soil (Figure 6). This indicated that seedling growth was stunted in mine tailings over the 12-month period, although not significantly different from seedling growth in grassland soil.

### 2.5. Soil Analysis

The analysis of composite soil samples from each treatment highlighted several key properties (Table 2). Tailings exhibited lower levels of exchangeable cations compared to other soils, indicating that it is nutrient-poor. Tailings also contained markedly less organic matter, which is essential to maintain soil fertility and structure. Furthermore, the electrical conductivity of the tailings was considerably higher than that of the control soil, suggesting elevated levels of salinity [31].

Particle size distribution analysis revealed that grassland soil and tailings contained the highest proportions of coarse particles and fine sand (sandy–loam texture). In contrast, the control and old field soils contained higher levels of clay (clayey–loam texture). The presence of both clay and silt suggests that these soils have finer textures, which may enhance their capacity to retain moisture and nutrients.

The control soil, with its balanced nutrient profile and absence of contaminants, served as a baseline for optimal plant growth. Grassland soils, characterized by lower nutrient content, resembled sandy, nutrient-poor substrates where *C. bulbispermum* is typically absent. Despite being affected by past agricultural use, old field soils aligned with the species’ preference for nutrient-rich, clay-based environments. Mine tailings, the harshest substrate tested, displayed extremely low nutrient levels and potential toxicity from heavy metals [32]. However, previous studies have shown that metal toxicity in mine tailings does not adversely affect *C. bulbispermum* [23].

## 3. Discussion

### 3.1. Species Distribution

*Crinum bulbispermum* displays a broad distribution across ancient, old-growth grasslands of eastern South Africa. This pattern aligns with the findings of Johnson and Raguso [33] on the distribution of hawkmoth-pollinated species. They noted that long-tubed species, including *C. bulbispermum*, are concentrated in the eastern regions of South Africa. This distribution is likely related to favorable ecological conditions, such as specific precipitation patterns and proximity to river systems, which create ideal habitats for survival and reproduction. The proximity of many *C. bulbispermum* populations to the Vaal River and other waterways suggests that hydrological patterns, such as seasonal flooding, water availability, and alluvial soil, play a critical role in the species’ life cycle and distribution.

The absence of *C. bulbispermum* populations from the western regions of South Africa can largely be attributed to aridity. Its restricted occurrence along the floodplains of the largest perennial river in the Northern Cape (Orange River) reflects hydrochory (water-mediated seed dispersal) and stable moisture availability in an otherwise low-rainfall region [34]. Understanding these distribution patterns is essential to gain insights into the species’ biology and its application in ecological restoration. Population densities in specific regions indicate ecological niche areas that define ideal habitat conditions for this species [35].

### 3.2. Habitat Suitability

The spatial overlap of *C. bulbispermum* populations with active mining areas in South Africa indicates that the natural habitat of this species is now surrounded by disturbed landscapes. Its persistence near mines suggests it is a native species inherently adapted to the local environmental conditions, including those found on tailings, making it a promising candidate for restoration. However, distribution gaps within heavily mined regions suggest displacement due to mining infrastructure, such as tailings dams and urban development. The distribution maps thus serve as a valuable tool to delineate the species’ historical biogeographical range and identify areas suitable for restoration.

A species distribution model highlighted suitable habitat in the Grassland Biome, particularly in Gauteng, Mpumalanga, and KwaZulu-Natal, which aligns with the species’ known ecological preferences [36,37]. This model, based on environmental variables, provided a more comprehensive understanding of the species’ ecological niche and identified additional areas where *C. bulbispermum* could potentially be established in mine restoration projects outside its natural range. This confirms it is a climatically and edaphically suitable geophyte for restoration initiatives on the Highveld [38].

Three key variables emerged as primary determinants in the SDM: vegetation type, annual precipitation, and proximity to rivers. Together, these accounted for over 80% of the model’s explanatory power, emphasizing their importance in defining suitable habitats. Furthermore, the permutation importance (measure of the variables’ influence on model accuracy) of annual precipitation exceeded 60%, highlighting the species’ strong dependence on moisture availability. Proximity to rivers also played a major role, consistent with the species’ preference for marshy or floodplain habitats. These findings are consistent with Bradie and Leung [39], that around 80% of SDMs are explained by precipitation and temperature, with proximity to water being a key factor in 42% of models.

The eastern region’s higher rainfall and favorable vegetation (grasslands) creates optimal conditions for *C. bulbispermum*. As a geophyte with a large underground storage organ, the species is tolerant of fire and cold and can resprout after above-ground parts are destroyed [17]. It allows for the plant to resprout after above-ground parts have been destroyed. Seasonal flooding creates ideal conditions for the species’ reproduction and establishment [40]. The recalcitrant seeds of *C. bulbispermum* have adapted for hydrochory (dispersal through waterways). These greyish-green, corklike seeds float easily and require marshy soil with constant moisture to germinate and establish. Seed germination must happen within three months or lose their viability [41]. These adaptations explain why dense populations occur along stream banks and why the seeds have the ability to establish in seasonally wet mine tailings.

In contrast, the arid western regions, such as the Northern Cape, exhibit low habitat suitability for *C. bulbispermum* [42], with the exception of areas along the seasonally flooded Orange River. The surrounding semi-desert vegetation and low rainfall create unsuitable conditions for the species [43], which do not support the moisture and soil requirements for the species. Research shows that SDMs often predict the absence of plant species from arid zones due to climatic limitations such as low precipitation and high temperatures [42,44].

### 3.3. Germination and Establishment

*Crinum bulbispermum* seeds achieved a germination rate of over 85%, consistent with the findings of Carpenter and Ostmark [45] for *Amaryllis* species, which reported a mean germination rate of 86%. This confirms the species’ germination ability and supports its potential inclusion in seed mixes for ecological restoration [46]. Seeds germinated equally well across different soil types, including chemically stressful mine tailings (Table 2). This resilience is attributed to the seeds having a high moisture content and hypogeal germination, which involves an embryonic stem being pushed down into the soil to form a small, subterranean bulb with roots. This strategy allows for germination above ground to presumably remain unaffected by conditions below. The evolutionary strategy of fewer, larger seeds increases the chance of survival [47], as this trait allows the embryo greater resource availability during germination, which increases the likelihood of rapid establishment and survival [48].

Seedlings established in all four soil treatments, including tailings, demonstrate the species’ potential for restoration. However, a reduction in productivity—measured by leaf length—was observed over time. There was no significant difference between seedling growth in mine tailings and grassland soil, but growth was notably stunted in both. This is likely explained by low nutrient levels and the poor physical structure of both these sandy soils [32]. The high salinity of tailings and limited nutrient-holding capacity of sandy textures suggest suboptimal conditions for long-term growth, indicating that *C. bulbispermum* is not ideally suited to saline, nutrient-poor environments without soil amendments.

Gold mine tailings, which are dominated by fine sand (coarse soil), exhibit rapid drainage and poor water retention. Such conditions challenge early seedling establishment due to reduced nutrient availability [49]. Although *C. bulbispermum* naturally prefers moist soils, it can survive in seasonally dry environments, even with lower nutrient levels. This is because it is morphologically adapted to store nutrients and water, which explains its persistence in tailings, albeit stunted with limited growth.

Soil organic carbon (SOC) is a key indicator of soil health, as it reflects microbial activity and the presence of decomposed organic matter that supplies nutrients to plants [50]. Fine-textured soils (clays) typically contain higher SOC due to the greater surface area of fine particles for organic matter adsorption [51,52]. High SOC levels (>5%) improve soil fertility by enhancing nutrient availability, water retention, and overall structure [53,54]. The lack of organic carbon in the sandy mine tailings suggests lower microbial activity and poor soil structure, which limits plant growth and development.

SOC also plays a crucial role in supporting soil microbial communities. Soils rich in organic carbon sustain larger, more active microbial populations, which facilitate nutrient cycling, enhance soil structure, and suppress pathogens [55,56]. Increasing SOC through compost or organic amendments can improve soil conditions for plant growth and should be considered in restoration strategies for degraded sites like mine tailings [57].

Despite these limiting soil conditions, *C. bulbispermum* can survive for 12 months in mine tailings, indicating a degree of resilience to chemically stressful environments. The elevated salinity in tailings could otherwise lead to osmotic stress and inhibit water uptake [58,59]. Survival under such conditions suggests that the species possesses some tolerance to salinity, though this ability might be enhanced further through soil mitigation [60]. However, the long-term effects of these conditions—beyond 12 months—on growth and reproductive success remain uncertain. Further long-term studies (e.g., over three years to include flowering and fruiting) are required to assess the species’ viability and performance in such challenging substrates [60].

## 4. Methods

### 4.1. Experimental Layout and Data Collection

#### 4.1.1. Distribution Data Sources

Occurrence data for *C. bulbispermum* were gathered from several South Africa herbaria, namely BLFU, JRAU, KMG, PRE, PRU, and PUC (Holmgren and Holmgren, 2001). Distribution records were obtained for 103 specimens, of which only 10 included precise georeferenced coordinates. To supplement the limited data, additional georeferenced records were sourced from BODATSA [61] and iNaturalist (www.inaturalist.org, accessed on 13 February 2023) research-grade observations, adding 162 localities. Field surveys undertaken in the North West Province during November and December 2022 contributed a further 10 localities. This yielded a final dataset of 182 precise georeferenced occurrences.

#### 4.1.2. Standard Germination Tests

A standard germination test (SGT) was performed to assess seed germinability. The experiment consisted of 10 pots (2 L each), each containing 20 seeds. Seeds were collected from uncontaminated areas near Potchefstroom and the NWU Botanical Garden on 25 November 2022. Seeds were placed on the soil surface to mimic natural conditions, using a typical garden potting mix (50% topsoil and 50% organic matter). Germination was defined as the emergence of the cotyledon and radicle through the soil surface [62]. The germination rate was defined as the proportion of seeds that germinated.

In a second SGT, four soil types were tested to evaluate germination under conditions relevant to restoration: control (garden potting mix), grassland (marshy sandy soil), old field (disturbed clayey soil), and gold mine tailings. Each treatment comprised four replicates (2 L pots), with 10 seeds per pot. Pots were arranged in a randomized design in a greenhouse, watered twice a week to field capacity, and monitored for a 12-month period. Watering was paused during the winter dormancy period (June–August). Seed trays were maintained under controlled day and night temperatures (25 °C) and photosynthetically active radiation between 600 and 800 µmol m^−2^ s^−1^. Germination was recorded weekly per pot for each treatment.

#### 4.1.3. Plant Establishment Trials

Seedlings obtained from the second SGT were monitored for establishment in each soil treatment over time. Seedling survival and the length of longest leaf (from base to apex) were recorded monthly from month 3 to month 12. The experiment was maintained under the same greenhouse conditions described for the SGTs.

#### 4.1.4. Soil Preparation

Grassland and old field soil was collected from METSI (Mooi River Ecosystem Trials for Scientific Investigations), 25 km northeast of Potchefstroom. Gold tailings were collected from roadside mine spoils between Orkney and Klerksdorp in North West Province, South Africa. A commercial potting mix (50% topsoil and 50% organic matter) served as the control. Composite soil samples were taken from each treatment group for chemical and physical analyses (Table 2). Samples were air-dried, ground, and passed through a 5 mm sieve to remove large particles and organic debris.

### 4.2. Data Analyses

#### 4.2.1. Spatial Distribution

Georeferenced localities were processed in ArcGIS software (version 10.8.2). Duplicate records were removed, and coordinate formats were standardized using the Hartebeesthoek 1994 Geographical Coordinate System. Localities were plotted on a base map of South Africa, with the Grassland Biome overlaid to provide ecological context. Additional layers depicting mining activity and agricultural fields were added to assess distribution relative to land transformation.

#### 4.2.2. Species Distribution Modelling

Species distribution modelling was conducted using MaxEnt (maximum entropy modelling), which is effective for presence-only data [63,64]. The 182 locality records of *C. bulbispermum* were used to estimate habitat suitability and assess the relationship between environmental factors and its occurrence [65]. Bioclimatic variables and Shuttle Radar Topography Mission (SRTM) elevation data were sourced from WorldClim https://www.worldclim.org/ (accessed on 13 February 2023). Slope and aspect were derived from elevation, and Euclidean distance layers were created for various river orders and coastal proximity. A vegetation layer was added from the South African National Biodiversity Institute, https://bgis.sanbi.org, (accessed on 13 February 2023). All layers were resampled to a 1 km resolution and converted to ASCII format for analysis. Of the 26 variables, only those with pairwise correlation coefficients < 0.6 were retained to avoid multicollinearity.

The default settings of Maxent [66,67] can lead to complex, potentially overfitted models. To address this, the ENMeval package in R version 4.0.2 was used for parameter optimization [68]. Model performance and complexity were determined using the Akaike Information Criterion (AICc), with potential overfitting measured by the AUC difference and a 10% training omission rate [69,70]. Optimal settings included a regularization multiplier of 2 and use of the hinge feature.

The final model was trained using 80% of occurrence records, with the remaining 20% reserved for validation. The model ran for 100 iterations with a subsample approach and random seed for reproducibility. All other parameters were kept at their default values. Model accuracy was evaluated using AUC (area under the Receiver Operating Characteristic (ROC) curve) and jack-knife tests of each variable, both individually and collectively. AUC values closer to 1 indicate higher model predictive performance; values around 0.5 indicate no predictive power. The model output was classified using the ‘10 percentile training presence logistic threshold’ (0.3068) to define suitable and non-suitable habitat classes. Suitable habitats were classified into five suitability classes using a Natural Breaks (Jenks) method, i.e., low to very high (Figure 3).

#### 4.2.3. Germination Trials

Differences in germination rates among the treatment groups were compared with one-way Analysis of Variance (ANOVA). Prior to analysis, data were tested for normality (Shapiro–Wilk test) and homogeneity of variances (Levene’s test). The treatment groups included ‘germination percentage’ and ‘soil type’. Germination percentage per pot was the dependent variable and soil type was the independent factor. A significance level of *p* < 0.05 was set for all tests.

#### 4.2.4. Establishment Trials

The effect of soil type on seedling establishment was compared with one-way ANOVA. Prior to the analysis, data were checked for normality. The soil types tested included ‘control’, ‘grassland’, ‘old field’, and ‘tailings’. Establishment rate per treatment was calculated as the percentage of seedlings surviving after 12 months. Leaf length served as a proxy for productivity.

Post hoc pairwise comparisons were performed using Bonferroni correction to control for Type I error. A significance level of *p* < 0.05 was applied.

#### 4.2.5. Soil Analysis

Composite soil samples were digested in 15 mL of nitric acid and covered and heated on a sand stove in a fume hood at ~95 °C. After cooling, 3 mL of 30% hydrogen peroxide was added, followed by 10 mL of a 3 N HCl. The mixture was again placed in a sand bath in the fume hood and left to reflux for an hour until cooling down to 25 °C. The mixture was then filtered through filter paper into a 50 mL volumetric flask. The flask was then filled to volume with deionized water, and the analysis of elements at North-West University proceeded using Inductively Coupled Plasma Mass Spectrometry (Agilent 7500 series, Agilent Technologies, Santa Clara, CA, USA), calibrated with a certified multi-element standard solution (PerkenElmer Pure, PerkinElmer Inc., Shelton, CT, USA). Soil organic carbon (%C) was quantified using a LECO carbon analyzer. Particle size was determined via hydrometer analysis.

## 5. Conclusions

This study evaluated the potential of *Crinum bulbispermum* for use in the ecological restoration of gold mine tailings on the Highveld of South Africa. The species exhibited a well-defined distribution pattern associated with the eastern Grassland Biome, particularly in regions with high annual precipitation, river proximity, and seasonally marshy soils. Species distribution modelling (SDM) confirmed these ecological preferences and revealed that suitable habitat overlaps with areas currently disturbed by mining, indicating a potential for targeted restoration interventions.

The germination trials showed high viability, with an average germination rate of 85% across all soil types, including chemically stressful gold tailings. The species’ hypogeal germination strategy, supported by large, moisture-rich seeds and early bulb development, enhances establishment success under a range of soil conditions. Pot trials confirmed 100% seedling survival in all treatments after 12 months, although seedling productivity was reduced in nutrient-poor, sandy substrates such as mine tailings and grassland soils. This reduction was likely due to poor soil structure, low organic carbon content, and increased salinity, all of which limit nutrient availability and water retention. Despite these limitations, *C. bulbispermum* showed notable persistence, highlighting its resilience and capacity for survival in disturbed environments.

Taken together, these findings provide a restoration profile for *C. bulbispermum* and support its inclusion in seed mixes aimed at restoring seasonally moist or riparian zones on gold mine tailings. Its ecological compatibility, high germination rates, and tolerance to stressful substrates make it a promising native geophyte for restoration, especially in areas often overlooked by grass-dominated approaches. However, due to observed growth limitations under saline and nutrient-poor conditions, its use should be paired with soil amendments to improve long-term establishment and productivity. Further long-term field trials are recommended to assess reproductive success and resilience beyond early seedling stages. This study contributes valuable ecological and practical knowledge for advancing more inclusive and effective restoration strategies in South Africa’s mined landscapes.

## Figures and Tables

**Table 1:** Percentage contribution and permutation importance of variables.

Variable Name	Variable Description	Percentage Contribution (%)	Permutation Importance
Veg	Vegetation type	39	24.6
bio 12	Annual precipitation	35.5	61.8
Distall	Distance to all rivers	13.9	6.4
bio 11	Mean temperature of coldest quarter	6.2	2.9
Slope	Slope	3	1.4
Disthigh	Distance to higher-order rivers	1.4	1.2
bio 14	Precipitation of driest month	0.5	1.2
Elev	Elevation	0.2	0
bio 3	Isothermality (mean diurnal range (mean of monthly max temp − min temp))/Temperature annual range (max temperature of warmest month − min temperature of coldest month) (×100)	0.2	0.5

**Table 2:** Soil chemical and physical properties for the different soil treatments (CS—control soil, GR—grassland, OF—old field, TS—tailings). CEC, cation exchange capacity; S-value, saturation index; BS, base saturation; EC, electrical conductivity; %C, percentage soil organic carbon; 10 ppb detection levels in soil digestates were achievable with ICP-MS but not validated.

Exchangeable Cations								
**Sample**	**Ca**	**Mg**	**K**	**Na**	**CEC**	**S-value**	**BS** (%)	**pH** (H_2_O)
			(cmol(+)/kg)					
CS	17.46	8.78	0.60	0.34	22.80	27.18	119.16	7.65
GR	15.64	5.00	0.49	0.17	17.78	21.30	119.90	7.26
OF	19.65	9.01	0.42	0.36	24.62	29.45	119.56	7.64
TS	1.26	0.33	0.04	0.03	1.37	1.65	120.85	7.22
Nutrient Status								
**Sample**	**Ca**	**Mg**	**K**	**Na**	**P**	**C** **(%)**	**EC**	**pH** (KCl)
			(mg/kg)				(mS/m)	
CS	3499.07	1067.21	234.92	77.83	24.23	4.67	83.25	7.82
GR	3133.57	607.16	192.93	40.07	9.86	4.91	111.50	7.93
OF	3937.80	1094.86	165.54	83.04	25.18	5.47	87.50	7.65
TS	252.05	39.83	15.09	6.53	24.99	0.11	194.50	7.79
Particle Size Distribution								
**Sample**	**>2 mm** **(%)**	**Very** **Coarse Sand**	**Coarse** **Sand**	**Medium** **Sand**	**Fine** **Sand**	**Very Fine**	**Silt**	**Clay**
						**Sand**		
CS	1.66	2.78	3.19	6.52	10.51	8.74	33.55	34.70
GR	34.15	6.58	5.09	11.33	23.29	17.47	26.39	9.86
OF	1.85	3.01	3.03	7.57	9.37	8.39	35.83	32.81
TS	29.48	9.26	7.46	8.62	25.69	33.57	12.77	2.63

## Data Availability

The data presented in this study are available on request from the corresponding author. The data are part of a master’s degree study and will not be publicly available until the dissertation is published. All dissertations and theses of North-West University are available at https://library.nwu.ac.za/theses-and-dissertations (accessed on 1 January 2025).

## Abbreviations

The following abbreviations are used in this manuscript: AUC—Area Under the Curve; BODATSA—Botanical Database of Southern Africa; CEC—Cation Exchange Capacity; EC—Electrical Conductivity; ENM—Ecological Niche Modelling; ICP-MS—Inductively Coupled Plasma Mass Spectrometry; NWU—North-West University; ROC—Receiver Operating Characteristic; SDM—Species Distribution Modelling; SGT—Standard Germination Test; SOC—Soil Organic Carbon; SRTM—Shuttle Radar Topography Mission; WA—Western Australia.
